# Adhesion Studies of CrC/a-C:H Coatings Deposited with Anode Assisted Reactive Magnetron Sputtering Combined with DC-Pulsed Plasma Enhanced Chemical Vapor Deposition

**DOI:** 10.3390/ma14112954

**Published:** 2021-05-30

**Authors:** Zhihong Huang, Zhijie Chen, Wenchang Lang, Xianghong Wang

**Affiliations:** Department of Mechanical Engineering, Wenzhou Polytechnic, Wenzhou 325035, China; 13600045268@126.com (Z.H.); chenzhijie5262@163.com (Z.C.); wolfwc@163.com (W.L.)

**Keywords:** a-C:H, interlayer, adhesion, anode assisted, magnetron sputtering, plasma-enhanced chemical vapor deposition

## Abstract

We studied the effect of CrC interlayers with different carbon contents on the adhesion of CrC/a-C:H coatings prepared by anode assisted reactive magnetron sputtering combined with DC-pulsed plasma enhanced chemical vapor deposition. The adhesion of the coating was measured by indentation and scratching. The coatings were characterized by Raman, XPS, SEM and Nanoindentation. The adhesion of the CrC/a-C:H coating is best when the carbon content in the interlayer of CrC is 44.5%, the scratch adhesion is 74 N, and the indentation adhesion is HF1. In this case, the elastic modulus of the interlayer CrC (284 GPa) is closest to that of the a-C:H layer (274 GPa). In conclusion, when there is no graphitization in the CrC interlayer, and the elastic modulus of the CrC interlayer is close to that of the a-C:H layer, the CrC/a-C:H coatings show the best adhesion.

## 1. Introduction

Diamond-like carbon (DLC) coatings are excellent solid lubricants as they lower friction, possess high hardness values and excellent wear resistance and chemical inertness [1,2,3]. Thus, DLC coating-based lubricants are widely used in various industrial applications including the automotive industry [4,5].

However, the bonding strength of DLC films on steel substrates is poor due to the significant differences between the films and the substrate. At present, the introduction of multiple gradient transition layers between the steel substrate and DLC film, to achieve gradual transition in composition and properties, is one of the most effective methods to improve the adhesion in the steel substrate/DLC system [6,7,8,9].

The amorphous carbon films can be divided into hydrogen-free carbon films (a-C) and hydrogenated carbon films (a-C:H) according to whether there is hydrogen in the composition [10]. Hydrogen-free carbon film (hardness of about 15 GPa) can be formed by sputtering a graphite target. In a magnetron sputtering device, a CrC/a-C coating without interface gradient transition can be formed by continuously reducing the power of the chromium target and increasing the power of the graphite target, so as to obtain good film substrate adhesion [11,12,13]. However, a hydrogenated carbon film (hardness of about 25 GPa) is formed by plasma enhanced chemical vapor deposition (PECVD) of methane or acetylene [14,15,16,17], and its adhesion is lower than that of the hydrogen-free carbon film prepared by magnetron sputtering. To obtain amorphous carbon films with high surface hardness and good adhesion, the CrC/a-C:H coating was prepared by magnetron sputtering combined with PECVD. Among them, a CrC gradient transition layer is selected as the intermediate layer, which has a low carbon content near the substrate side and a high carbon content near the a-C:H layer. It is well known that the optimal gas pressure and bias voltage of the magnetron sputtering process is about 0.5 Pa and 100 V, while that of PECVD amorphous carbon film is about 1.0 Pa and 1000 V. Therefore, the transition from magnetron sputtering to the PECVD process should not adopt a gentle change of process parameters, but should adopt an abrupt change. At the same time, according to the general rule of gradient coating design, a carbon content as high as possible is required outside the CrC transition layer to cooperate with the top amorphous carbon film. However, to avoid the target poisoning range in the reactive magnetron sputtering process, the carbon content outside the CrC layer must be significantly lower than that of the top a-C:H layer. In the process of depositing the CrC/a-C:H coating by magnetron sputtering combined with PECVD, the process parameters and the composition of the coating change suddenly, which makes the interlayer bonding of CrC/a-C:H coating very sensitive to the change of process parameters. Therefore, the design of the interlayer structure and the process parameters in control of deposition are of great significance to obtain high hardness and good adhesion of the CrC/a-C:H coating, however, little research has been done in this field.

To obtain a hard and wear resistant metal carbide interlayer, high ion current densities at the substrates were necessary. Usually closed field unbalanced magnetron sputter (CFUBMS) technology [18,19,20,21] is employed to prevent electric escape and improve the ionization rate of sputter atoms. In addition, an innovative method to improve the ionization rate is to use the central anode developed by Wolf-Dieter Münz [22], by which the sputtering DLC coatings with 40–55 GPa hardness and low friction in the 0.05–0.07 range, were deposited onto threefold rotated stainless-steel substrates.

In this work, DLC coating is deposited with a hybrid process which consisted of sputter deposition of a CrC interlayer followed with a DC-pulsed PECVD deposition of a pure a-C:H top layer. Different from CFUBMS and the central anode method, in the sputter process, the discharge is established between the sputter cathode and a specialized anode, and the chamber has a −70 V bias from the anode. Such an auxiliary anode arrangement increases the difficulty of electron escape compared with the traditional sputtering in which the chamber is used as the anode. Denser plasma can be obtained, and the bias current is three to four times greater than that of conventional sputtering, so a denser coating is predictable. This anode-assisted magnetron sputter method is very suitable for coating applications that require a high coating density and the substrate is not sensitive to temperature, such as tool, mold and component coatings.

This work reports the effect of C content in the CrC interlayer on the adhesion of CrC/a-C:H film. We also analyzed the properties of a single CrC layer and multi-layered CrC/a-C:H structures to understand the interaction between the interlayers.

## 2. Materials and Methods

### 2.1. Film Preparation

**Figure 1:**
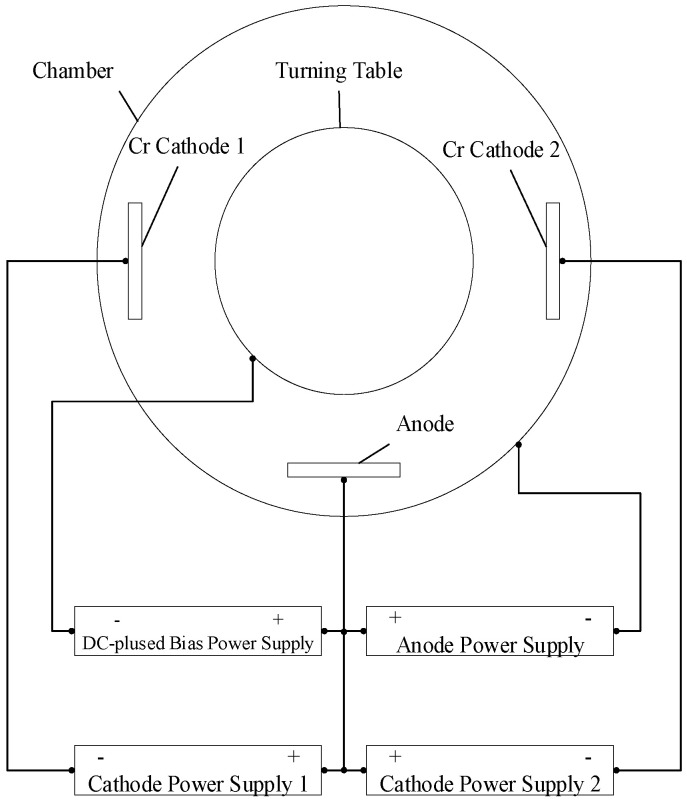
Schematic diagram of magnetron sputter equipment.

CrC and CrC/a-C:H films were prepared using a homemade PVD coating system. The dimension of the vacuum chamber was 800 mm in diameter and 900 mm in height, and the effective coating area was 650 mm in diameter and 500 mm in height. Two 600 × 120 mm^2^ chromium targets (99.95% pure) were positioned as the cathode. A water-cooled copper plate with the same size as the cathode was used as the anode. Three Advanced Energy Pinnacle 20K DC power supplies and a Pinnacle plus 10K DC-pulsed power supply were connected to the system (see Figure 1).

The distance between targets and the substrate was 80 mm. The coating was deposited on Si (100) wafer (for composition and structure characterization) and M2 high speed steel (for the characterization of mechanical properties) with an HRC64 hardness and 0.8 μm roughness (Ra). Along with the test pieces, dummy loads were installed so that the discharge power of the PECVD process could reach about 1.0 kW. The coating device operates in the following order: heating, plasma etching, deposition of CrC interlayer, deposition of a-C:H layer and cooling. The vacuum during the heating step was 5.0 × 10^−3^ Pa. It was achieved by turbo molecular pump with a nominal pump speed of 2300 L/min, and the heating temperature was kept at 180 °C to eliminate water and air from the chamber. Plasma etching was performed in Ar-H_2_ plasma for 30 min. For the deposition of the CrC layer, Ar was the working gas, C_2_H_2_ gas was the reaction gas, and the flow rate of C_2_H_2_ was set to 0–30 sccm. In order to obtain gradient transition, a pure Cr layer was deposited first, and then C_2_H_2_ flow was gradually increased until the set value was maintained. The deposition time of the pure Cr layer, gradient layer, and CrC layer were 10, 20, and 10 min, respectively. In order to express them with convenience, the CrC films deposited by different C_2_H_2_ flows were successively marked as CrC0, CrC5, CrC10, CrC15, CrC20, CrC25 and CrC30. The a-C:H film was deposited with DC-pulsed PECVD. The bias voltage was −600 V with a frequency of 250 kHz and duty cycle of 40%. The deposition time for a-C:H film was 2 h (see Table 1).

### 2.2. Film Characterization

Data on sample chemical compositions were obtained using X-ray photoelectron spectroscopy (ESCALAB 250×, Thermo Fisher, E. Grinstead, UK) with monochromatized Al Ka radiation. Prior to the analysis, the sample surfaces were cleaned with a 4 keV Ar^+^ flow. The Raman spectrum was measured by using a Renishaw RM-1000 spectrometer (Renishaw, Gloucestershire, UK) equipped with a 514.5 nm Ar-laser. Field emission scanning electron microscopy (FESEM, Zeiss sigma HD, Oberkochen, Germany) was used to characterize the cross-section morphology and measure the coating thickness. The nanoindentor (Hysitron TI950, Billerica, MA, USA) in the Nano Dynamic Mechanical Analysis mode measured the micro-hardness. Six tests were performed for each sample on different sample sections. The bonding force between the substrate and the coating was measured by Anton Paar Revetest film scratch tester (Wundschuh, Australia). The specific parameters are as follows: diamond indenter radius 0.2 mm, starting load 1 N, ending load 100 N, loading rate 99 N/min, scratch speed 3 mm/min, scratch length 3 mm. According to VDI standard 3198, the coating adhesion was tested qualitatively. A diamond cone with a taper of 120 degrees and a fillet radius of 0.1 mm was pressed into the coated metal substrate with a load of 150 kg. The cracks around the indentation and the peeling off of the coating were observed with a microscope, and the adhesion grade was confirmed by comparing with the standard pictures.

## 3. Results and Discussion

**Figure 2:**
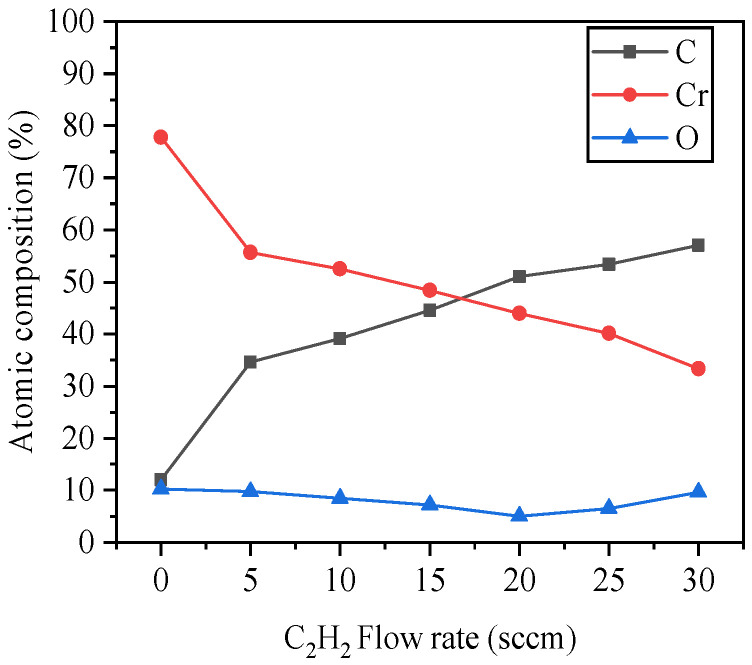
Composition of CrC coatings with increasing C_2_H_2_ flow rate.

XPS spectra of the CrC films identified Cr, C and O presence (see Figure 2). As the C_2_H_2_ flow rate increased from 0 to 30 sccm, the C content of CrC samples increased from 12 to 57% while the Cr content decreased from 78 to 33%, and the O content remained below 10%. The CrC0 sample is designed as a pure Cr layer, but it contains 12% carbon and 8% oxygen according to XPS composition analysis. It may be that the sample was polluted by exposure to the environment. Moreover, as a calibration element of XPS analysis, C is an almost inevitable impurity.

**Figure 3:**
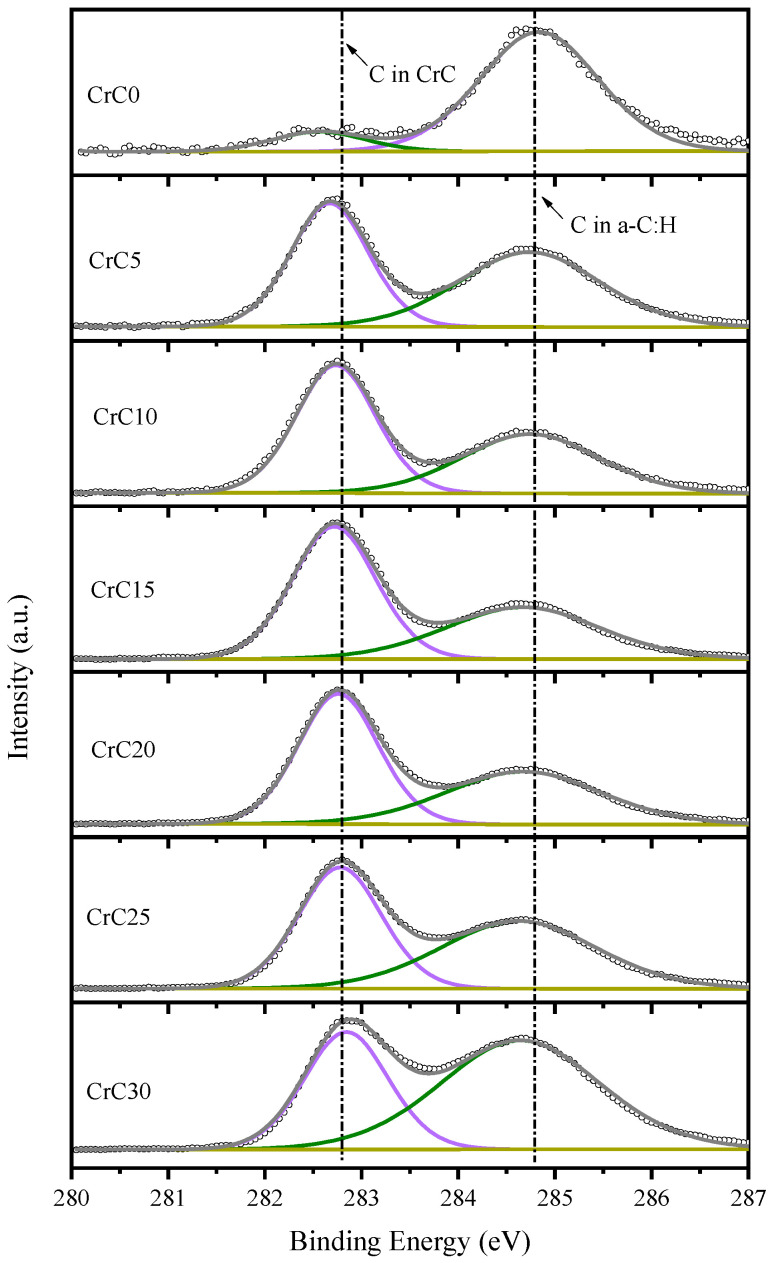
C1s XPS spectra of CrC films deposited at different C_2_H_2_ flow rates.

High-resolution C1s XPS spectra of CrC films deposited at different C_2_H_2_ flow rates revealed carbide peaks at 282.8 eV and carbon peaks at 284.8 eV (see Figure 3). There is only a carbon peak in the C1s XPS spectrum of the CrC0 sample, which shows that the C element of the CrC0 sample is surface contamination and does not bond with Cr. There are carbon peaks and carbide peaks in C1s XPS spectra of other samples.

**Figure 4:**
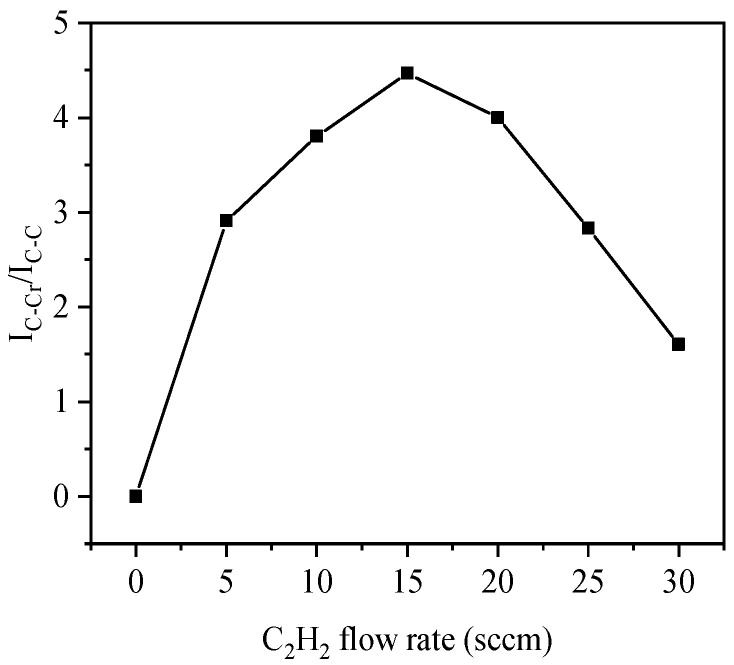
The atomic ratio of carbide to carbon in CrC coatings deposited at different C_2_H_2_ flow rates, carbide is C in CrC, carbon is C in a-C:H.

The results of peak fitting of C1s XPS spectra are shown in Figure 4. As the C_2_H_2_ flow rate was increased, the proportion of carbide first grows and then decreases. At 15 sccm, the proportion of carbide is the largest. When C_2_H_2_ is lower than 15 sccm C_2_H_2_ reacts with Cr to form CrC leading to an increase in carbide. When C_2_H_2_ exceeds 15 sccm amorphous carbon forms and the proportion of carbide deceases. When Thomas Zehnder [23] deposited a nano composite TiC/a-C: H coating by reactive PVD, it was observed that the carbide content first increased and then decreased with the increase of total carbon content. He divided the TiC/a-C: H coating into three regions: metal rich region, carbide rich region, and a-C: H region.

**Figure 5:**
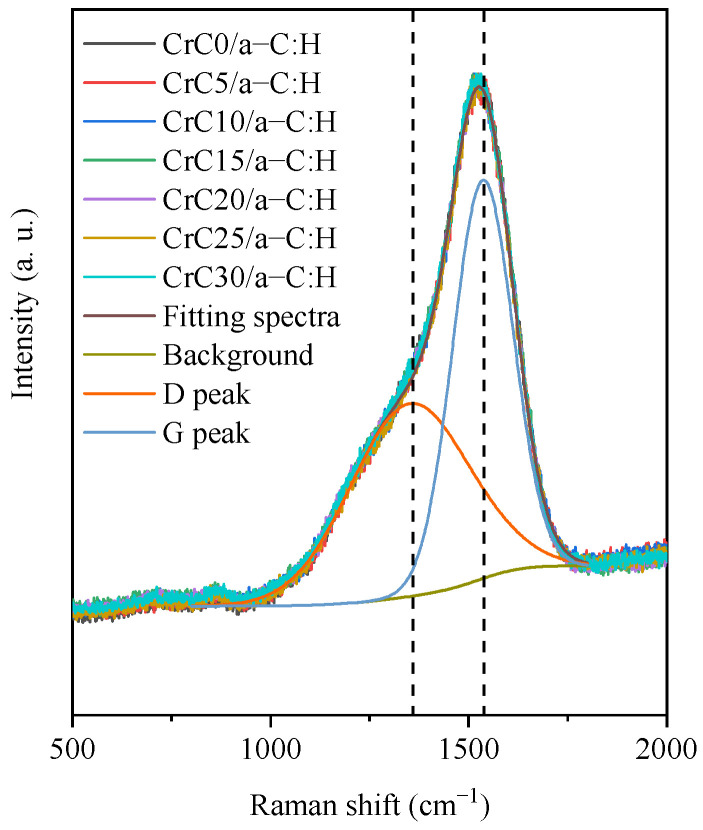
Normalized Raman spectra of CrC/a-C:H coatings deposited at different C_2_H_2_ flow rates.

Raman spectra of the CrC/a-C:H films showed the typical asymmetric inclined patterns typical for amorphous carbon [14,24] (see Figure 5). The seven Raman scattering spectra of the CrC/a-C:H coating with different C content as interlayers are basically coincident. The CrC/a-C:H film spectrum deposited by different interlayers of the CrC and the same top layer a-C:H process have the same Raman spectrum, which indicates that the process parameters of the CrC layer do not affect the structure of the a-C:H coating. The D peak and G peak of the Raman spectra of CrC0/a-C:H coatings are determined using Gaussian fitting [24,25,26]. The D peak position at 1346.9 cm^−1^ belongs to sp^2^ C stretching. The G peak position at 1535.5 cm^−1^ corresponds to the breathing mode of sp^2^ in the rings. The intensity ratio of the D peak and G peak (I_D_/I_G_) is 0.9.

**Figure 6:**
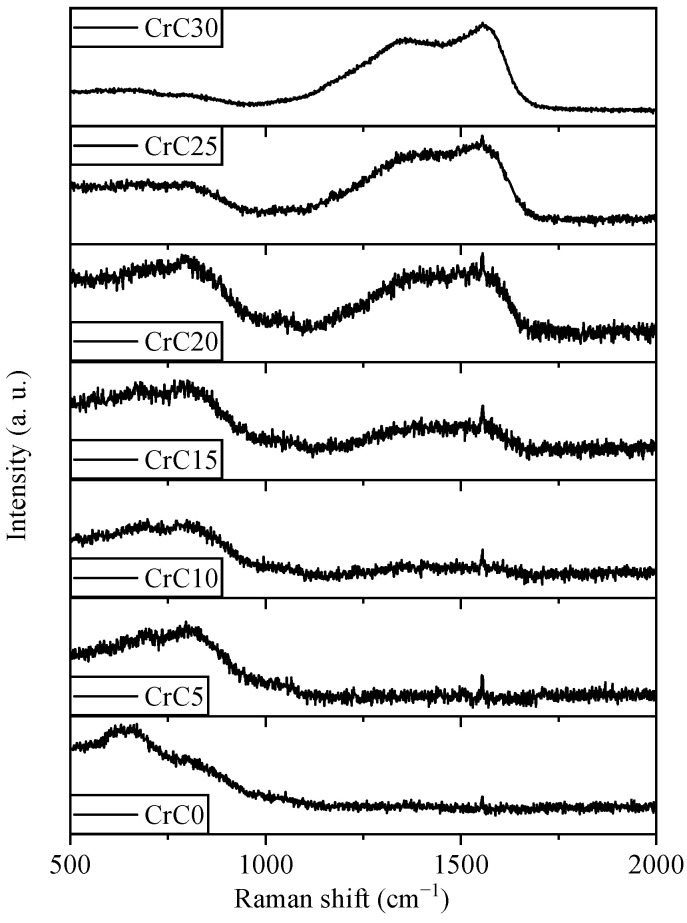
Normalized Raman spectra of CrC coatings deposited at different C_2_H_2_ flow rates.

Raman spectra of the CrC0, CrC5 and CrC10 films did not reveal the presence of the D and G peaks of amorphous carbon (see Figure 6), which indicates that the adjacent positions of carbon atoms in these CrC coatings were all Cr atoms, which could only combine with Cr to form C-Cr bonds.

The characteristic peaks of amorphous carbon appear in CrC15, CrC20, CrC25 and CrC30 coatings. By comparing the Raman scattering intensity of each sample at 500–1000 cm^−1^ and 1200–1800 cm^−1^, it is found that the graphitization characteristics of CrC coating are more obvious with the increase of carbon content. It shows that there are surplus carbon atoms in CrC15, CrC20, CrC25 and CrC30 coatings, which combine with each other to form C-C bonds. It is worth noting that samples CrC0, CrC5, and CrC10 have C-C bonds in XPS, but not in Raman spectra. The reason is that the contaminated carbon only exists on the surface of the sample, and the effective detection depth of Raman is deeper than that of XPS.

**Figure 7:**
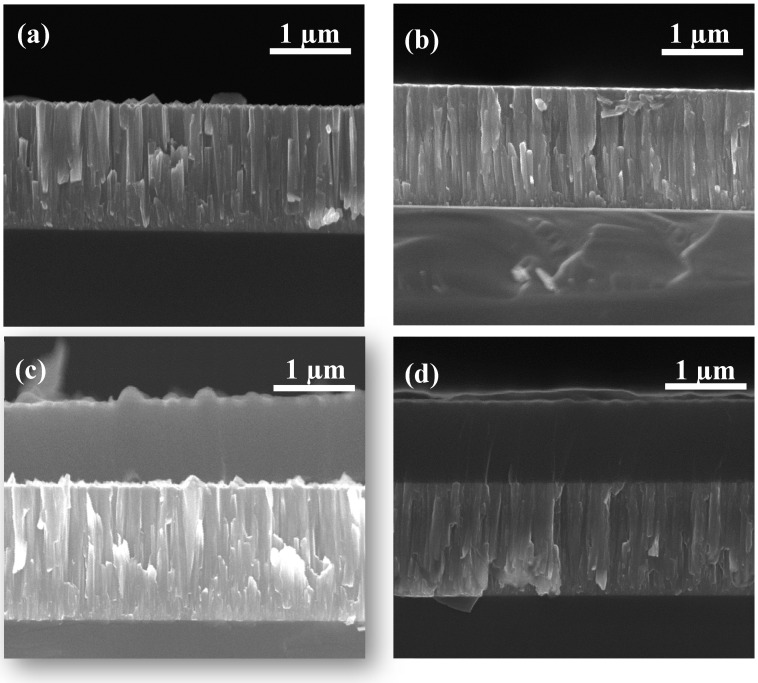
SEM images of CrC (**a**,**b**) and CrC/a-C:H (**c**,**d**) coatings deposited on Si substrate deposited at 0 (**a**,**c**) and 30 (**b**,**d**) sccm C_2_H_2_ flow rates.

According to the SEM observation, the thickness of the CrC coating deposited at different C_2_H_2_ flow rates is about 1.3 µm, and the thickness of the CrC/a-C: H coating is about 2.3 µm, including the 1.4 µm CrC layer and 0.9 µm a-C:H layer. Figure 7a,b shows cross-sectional SEM photos of CrC0 and CrC30 coatings, respectively. The CrC coating has a columnar crystal structure, because of the lattice mismatch between Si and Cr, the crystal size of the CrC coating near the Si-C interface is fine, while the grain size at the top of the CrC coating is large. This phenomenon is in line with the growth law of heterogeneous films. The top profile of the CrC0 coating is zigzag, and the top profile of the CrC30 coating is a smooth straight line, which indicates that the CrC30 coating had some amorphous characteristics compared with CrC0. This change of the morphology from columnar to fully dense was also in the literature; other CrC films had a 25–85 % content of C [27]. High C causes the crystalline transformation to an amorphous one, which, in turn, forces the carbon matrix grain size to decease [28].

SEM images of CrC0/a-C:H and CrC30/a-C:H section coatings showed that CrC/a-C:H coatings contain a columnar CrC and an amorphous a-C:H layer (see Figure 7c,d). There is a sharp columnar/amorphous interface in the CrC0/a-C:H coating, while the a-C:H layer grew epitaxially on the CrC layer in the CrC30/a-C:H coating, which made the corresponding interface more compact [29].

**Figure 8:**
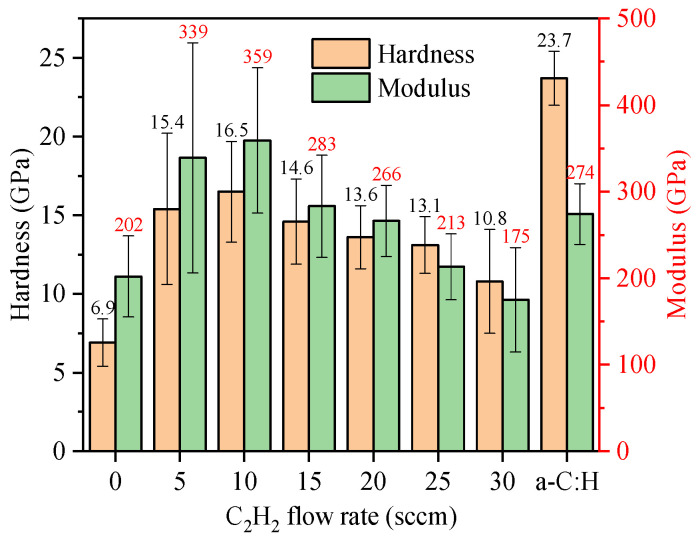
Hardness and modulus of CrC coatings with increasing C_2_H_2_ flow rate.

Hardness of the Cr (CrC0) coating was equal to 7.9 GPa (see Figure 8). As the C_2_H_2_ flow rate was increased, the CrC film hardness of CrC films increased (reaching the highest value equal to 16.5 GPa at 10 sccm C_2_H_2_ flow) and then decreased to 10.8 GPa. The elastic modulus of the CrC films increased with the C_2_H_2_ flow rate from 202 to 359 GPa but then decreased to 175 GPa. Anderson et al. observed a similar decrease of 10.6 to 6.9 GPa for the non-reactively sputtered CrC films containing 25–85 % C [27]. Such low hardness values of these films were related to a “two-phase” region, sp^2^-rich a-C:H and amorphous CrC_x_ phases, which were earlier detected by Raman and XPS. The hardness decreased because of high sp^2^ carbon content. However, Gassner et al. reported 20 GPa hardness for film containing sputtered CrC nanoparticles embedded in an a-C:H matrix [30]. Because our values are in between these literature ones, we concluded that our CrC films were indeed embedded in an sp^2^-rich matrix.

However, further analysis is needed for the determination of CrC film phases. The hardness and the elastic modulus of the CrC/a-C:H film were equal to 23.7 ± 1.7 GPa and 274 ± 35 GPa, respectively. Since the surface layers of these films were deposited with the same parameters, no significant hardness of modulus fluctuations were anticipated.

**Figure 9:**
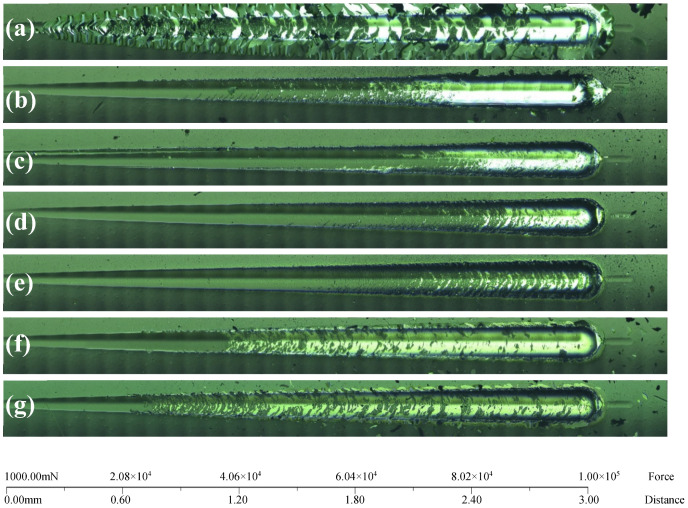
Scratch images of the CrC/a-C:H coatings with the CrC interlayer deposited at (**a**) 0, (**b**) 5, (**c**) 10, (**d**) 15, (**e**) 20, (**f**) 25, and (**g**) 30 sccm C_2_H_2_ flow rates.

Figure 9 shows the scratch photos of CrC/a-C:H coating, and Figure 9a–g correspond to the CrC/a-C:H coating with a flow rate of 0, 5, 10, 15, 20, 25 and 30 sccm, respectively. According to the peeling off of the coating on the outer edge of the scratch in the photo, it can be divided into three categories: a large peeling off, partial peeling off, and no peeling off. A lot of spalling occurred at the outer edge of the scratch of the CrC0/a-C:H coating (Figure 9a) from the beginning of loading, which indicates that the adhesion between the a-C:H coating and the substrate could not be improved by using a pure chromium layer as the adhesive layer. Because the pure Cr layer does not contain carbon, the combination of Cr and a-C:H will lead to the abrupt change of carbon potential. The affinity between Cr and a-C:H was not as good as that of CrC.

The outer edge of CrC25/a-C:H and CrC30/a-C:H coatings peeled off slightly, and the peeling of the CrC30/a-C:H coating was earlier than that of the CrC25/a-C:H coating. The results show that the higher the content of C in the interlayer of CrC, the worse the adhesion. Raman and XPS analysis show that the CrC in the interlayer of the CrC25/a-C:H and CrC30/a-C:H coating has a graphite component. Because graphite can release stress through sliding when loading, the bearing capacity of graphitized CrC is poor. The higher the degree of graphitization of CrC, the worse the adhesion of the CrC/a-C:H coating.

No peeling off occurred on the outer edge of the scratch of CrC5/a-C:H, CrC10/a-C:H, CrC15/a-C:H and CrC20/a-C:H coating samples. In the four samples, with the increase of load, cracks first appear inside the scratch of the CrC5/a-C:H coating sample at about 27.9 N, and unconnected spalling occurs first in the CrC10/a-C:H coating sample at about 17.3 N. CrC15/a-C:H and CrC20/a-C:H coating samples show good adhesion, and they all have smooth scratch edges. Moreover, cracks, discontinuous peeling off and continuous peeling off occur above 40 N. The internal continuous peeling of the CrC20/a-C:H coating sample scratch at 60 N is earlier than that of the CrC15/a-C:H coating sample at 50 N. Therefore, the order of coating adhesion from good to bad can be roughly judged by observing the peeling state of coating around the scratch: CrC15/a-C:H > CrC20/a-C:H > CrC10/a-C:H > CrC5/a-C:H > CrC25/a-C:H > CrC30/a-C:H > CrC0/a-C:H.

**Figure 10:**
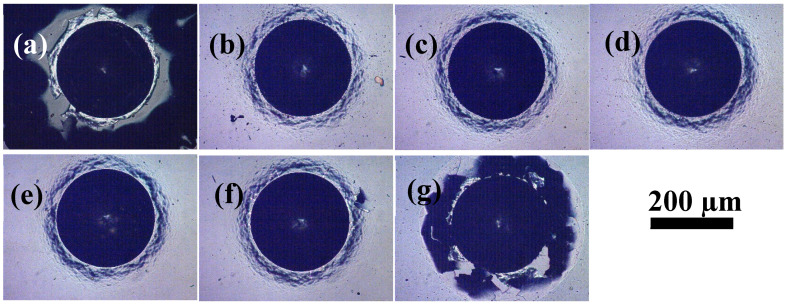
Indentation adhesion analysis of CrC/a-C:H coatings. The CrC interlayer was deposited with the C_2_H_2_ flow rate of (**a**) 0, (**b**) 5, (**c**) 10, (**d**) 15, (**e**) 20, (**f**) 25, and (**g**) 30 sccm.

Figure 10 shows the indentation photos of the CrC/a-C:H coating, and Figure 10a–g corresponds to the CrC/a-C:H coating with a flow rate of 0, 5, 10, 15, 20, 25 and 30 sccm, respectively. In the indentation adhesion test, a diamond indenter is pressed into the coating sample, and cracks and peeling off at the edge of the indentation due to stress release are observed. According to the VDI standard 3189, indentation adhesion is divided into six grades: HF1–HF6. It can be seen from the indentation photos that there is a large area of continuous peeling along the outer edge of the indentation of CrC0/a-C:H and CrC30/a-C:H coating samples, with the adhesion grade of HF6. There is radiated fibrous peeling at the indentation of the CrC25/a-C:H coating sample, with the adhesion grade of HF3. There is spot peeling at the indentation of the CrC5/a-C:H and CrC10/a-C:H coating samples, and the adhesion grade is HF2. For the CrC15/a-C:H and CrC20/a-C:H coating samples without peeling, the adhesion grade is HF1.

Further observation of the color of the bottom layer around the indentation shows that the interface of the CrC30/a-C:H coating is grayish black after peeling, which is lighter than the black of the a-C:H layer, and darker than the bright white of the metal substrate. It shows that the coating peeled from the interface between the middle layer of the CrC layer and the top layer of the a-C:H layer, and the appearance of the coating is the CrC layer. For the CrC0/a-C:H coating samples, the interface after peeling is bright white. Because the intermediate layer of the CrC0/a-C:H coating is the pure Cr layer, and the color of the pure Cr layer is similar to that of the base layer, the chemical composition of the interface material cannot be identified by the color peeling off. The composition of interface material can be judged by the color change of the displacement reaction.

CuSO_4_ + Fe→FeSO_4_ + Cu↓ (1)

Due to the corrosion potential of Cr > Cu > Fe, when there is a metal substrate at the stripping position, the main alloy element iron in the base material can replace copper in a CuSO_4_ solution and turn it red. When the stripping position is at the Cr layer, the displacement reaction will not occur. The CuSO_4_ solution was dripped on the indentation peeling place of the CrC0/a-C:H coating sample, and then it was cleaned after standing for one minute, and the peeling part did not change color, which indicated that the CrC0/a-C:H coating had peeled off from the interface between the middle chromium layer and the top a-C:H coating. According to the indentation peeling condition of the CrC0/a-C:H and CrC30/a-C:H coatings, it is easy to peel off from the interface between the intermediate layer and the top layer, rather than from the interface between the intermediate layer and the metal substrate.

The critical loads of scratch according to ISO 20,502 and the indentation adhesion class according to VDI standard 3189 of the CrC/a-C:H coating samples are listed in Table 2.

There are two interfaces in the CrC/a-C:H coating, the substrate/CrC layer, and the CrC layer/a-C:H layer; the bonding between the CrC and a-C:H layer is the weakest. When the coating is deformed, the stress is concentrated on the interface of the CrC and a-C:H layer, and the disharmony of the deformation causes the peeling and cracking of the coating. By adjusting the carbon content of the CrC layer, the elastic modulus of the CrC layer can be close to that of the a-C:H layer. So, during deformation, because the stress is no longer concentrated on the interface, but distributed in the whole transition zone, it is difficult for the crack to form and expand. Under this condition, the coating has the best adhesion.

## 4. Conclusions

In this paper, CrC/a-C:H coatings with different C contents were deposited on high-speed steel and silicon substrates by anodic assisted magnetron sputtering combined with PECVD. The C content was adjusted by changing the C_2_H_2_ flow rate during the deposition. The change of structure and mechanical properties of CrC layers with different carbon contents and the influence on the adhesion of the CrC/a-C:H coating have been studied. The results are as follows:

(1) The hardness and elastic modulus of the CrC coating first increased and then decreased as the C_2_H_2_ flow rate was increased. At the C_2_H_2_ flow rate equal to 10 sccm, the hardness and elastic modulus of CrC coatings were equal to 16.5 and 359 GPa, respectively.

(2) According to XPS and Raman analysis, higher C_2_H_2_ flow rates correlated with higher degrees of graphitization, while the hardness and elastic modulus of the coatings exhibited an opposite trend.

(3) When the elastic modulus of the CrC layer and the a-C:H layer are close, the coating has better adhesion, namely, 74 N by scratch test and HF1 by indentation test.

## Figures and Tables

**Table 1:** CrC and a-C:H film depositin parameters.

Process Parameters	Unit	CrC	a-C:H
Pressure	[Pa]	0.3	1.2
Ar flow	[sccm]	Pressure control	-
C_2_H_2_ flow	[sccm]	0–30	Pressure control
Cathode power	[W/cm^2^]	10	-
Bias Voltage	[V]	−100, 250 kHz, 1.2 µs	−600, 120 kHz, 1.2 µs
Time	[s]	2400	7200

**Table 2:** Adhesion classes and critical loads of the CrC and CrC/a-C films.

Coating	Adhesion Class	Lc1 [N]	Lc2 [N]	Lc3 [N]
CrC0/a-C:H	HF6	-	1.1	5.6
CrC5/a-C:H	HF2	27.9	20.3	56.0
CrC10/a-C:H	HF2	-	13.7	68.1
CrC15/a-C:H	HF1	43.4	41.0	74.0
CrC20/a-C:H	HF1	48.7	58.3	73.9
CrC25/a-C:H	HF3	-	21.6	34.1
CrC30/a-C:H	HF6	-	15.2	22.4

## Data Availability

All data is contained within the article.
